# Malignant Peripheral Nerve Sheath Tumor of the Small Bowel: A Case Report and Review of the Literature

**DOI:** 10.7759/cureus.75189

**Published:** 2024-12-05

**Authors:** Kleanthi - Christina Ampntin, Nikolaos Tasis, Maria Arnaouti, Maria Chrysi, Dimitrios P Korkolis, Aris Plastiras

**Affiliations:** 1 Department of Surgical Oncology, General Anticancer and Oncology Hospital of Athens "Saint Savvas", Athens, GRC; 2 Department of Pathology, General Anticancer and Oncology Hospital of Athens "Saint Savvas", Athens, GRC

**Keywords:** bowel cancer, malignant peripheral nerve sheath tumor (mpnst), malignant schwannoma, neurofibromatosis 1 (nf1), primary malignant peripheral nerve sheath tumor, small bowel mass

## Abstract

Malignant peripheral nerve sheath tumor (MPNST) is an uncommon type of sarcoma that arises from a peripheral nerve or any tissue with nerve sheath differentiation. It does not have any specific symptoms and shows great variability in clinical and radiological findings. It is usually associated with neurofibromatosis type 1 (NF1). Malignant peripheral nerve sheath tumors mostly originate from soft tissue organs and rarely originate from the small bowel. Less than 20 cases of intestinal MPNSTs are recorded worldwide. Therefore, we present this unique case of a 78-year-old woman, with no history of NF1, who presented with abdominal pain and vomiting and was diagnosed postoperatively with MPNST in the small bowel. Prompted by this case, we reviewed the literature for cases of intestinal MPNSTs.

## Introduction

Malignant peripheral nerve sheath tumor (MPNST) is a rare type of sarcoma. It accounts for approximately 5%-10% of all sarcomas and arises from a peripheral nerve or any tissue with nerve sheath differentiation [1]. Malignant peripheral nerve sheath tumors mostly originate from soft tissue organs in the trunk, extremities, or head and neck [1, 2]. Pathological reports like malignant schwannoma or neurofibrosarcoma have also been used in the past to describe these tumors [3]. It should be noted that the tumor is either sporadic or, more commonly, may be associated with neurofibromatosis type 1 (NF1), as about 25% to 50% of people with MPNST also have NF1 [3-7]. The diagnosis is challenging as MPNST does not cause any specific symptoms. The preoperative diagnosis is extremely difficult, as MPNSTs show great variability in clinical and radiological findings, and only immunohistochemistry of the final specimen can provide a definite diagnosis [3-7]. To our knowledge, there are fewer than 20 cases of MPNST in the gastrointestinal tract with no NF1, most of them concerning the small intestine [2-8], and, hereby, we present a rare case of a 78-year-old woman with MPNST in the small bowel who had no symptoms suggestive of NF1, who presented with a non-specific abdominal pain and vomiting as her primary symptoms. The diagnosis was made postoperatively after the pathological report. Prompted by this case, we conducted a review of the literature on other cases of intestinal MPNSTs and their management. 

## Case presentation

**Figure 1:**
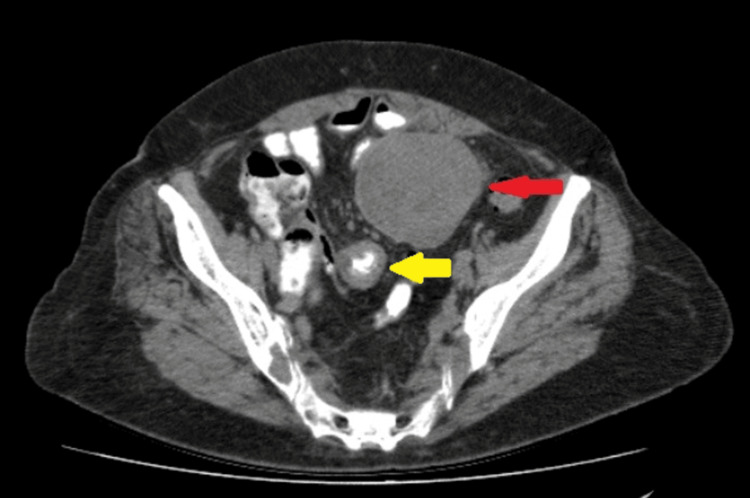
The CT scan (transverse view) showing a lesion (red arrow) and bowel wall thickening (yellow arrow).

**Figure 2:**
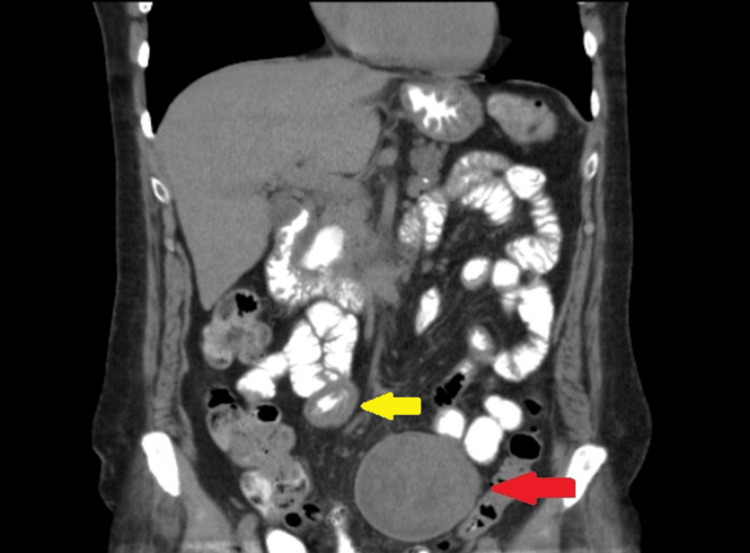
The CT scan (coronal view) showing a lesion (red arrow) and bowel wall thickening (yellow arrow).

A 78-year-old woman presented in our hospital complaining about solid stool and gas suspension for three days, diarrhea for a week, and non-specific pain in the lower abdomen with multiple episodes of vomiting during the last three months. Regarding her medical history, hypothyroidism and hypercholesterolemia were noted, with no allergies, smoking, or alcohol intake. Her surgical history included arthroplasty of both hips and a total hysterectomy 26 years ago due to endometrial cancer. She had no significant family history as well. Upon her presentation, vital signs were normal apart from mild increased heart rate. Physical examination revealed abdominal tenderness periumbilically and in the lower abdomen, and a palpable mass in the lower abdomen was noted. Laboratory blood tests, which included inflammatory markers, carcinoembryonic antigen (CEA), and cancer antigen 19-9 (Ca 19-9), were within normal rates. The patient underwent a contrast-enhanced computerized tomography (CECT) scan, which revealed a large mass in her abdomen and small bowel distention. Specifically, it showed a well-defined mass of mixed texture (with solid and cystic components) in the left lower abdomen whose dimensions were 7.1 x 7.3 cm. In addition, the CT scan revealed an adjacent abnormal small bowel wall thickening (Figures 1, 2). Following admission to the surgical clinic, a nasogastric and foley catheter were placed, and the patient underwent fluid resuscitation and monitoring.

**Figure 3:**
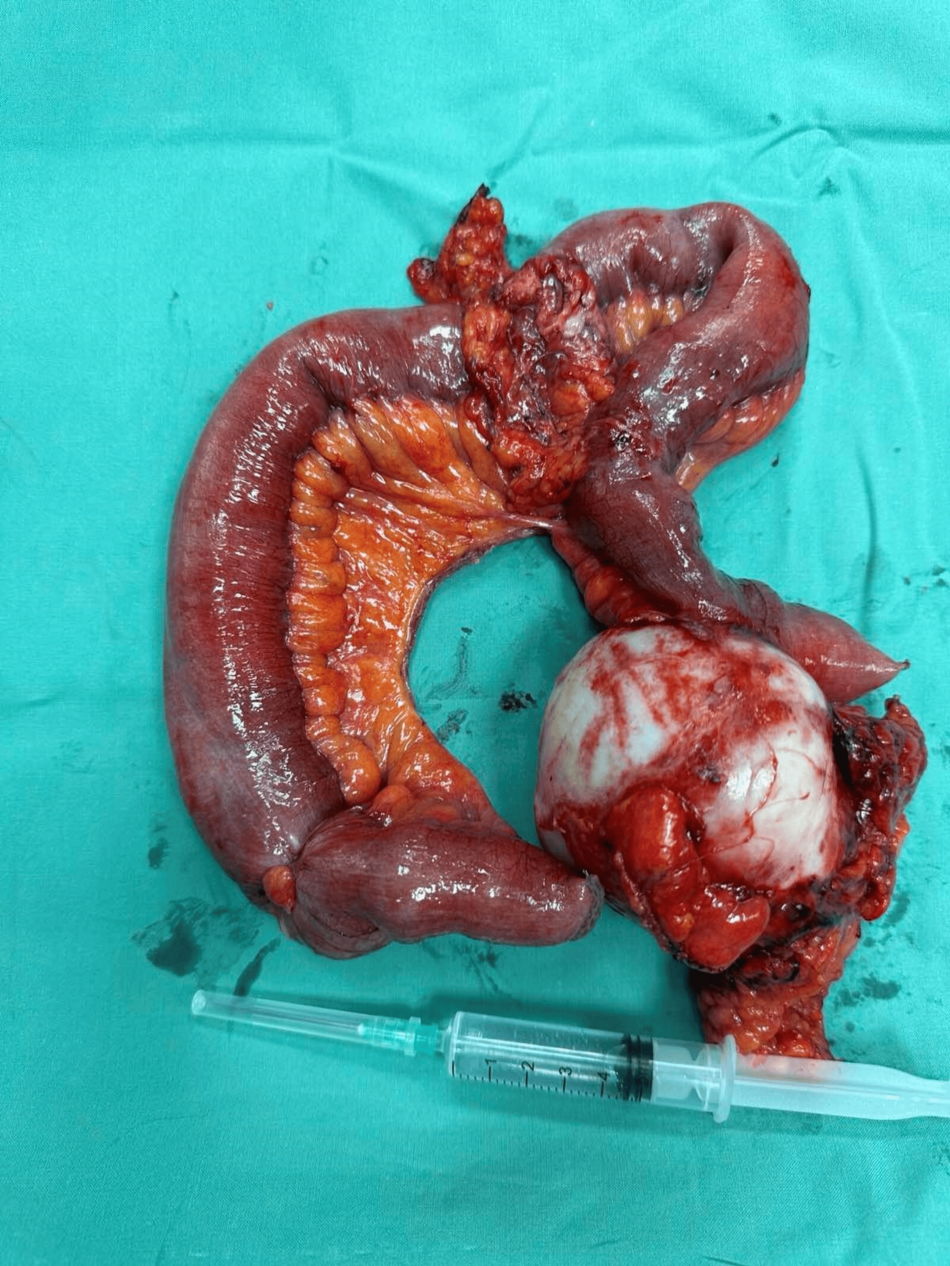
Surgical speciment of the small bowel segment including the masses

Due to the patient’s deteriorating abdominal pain, exploratory laparotomy was decided. Under general anesthesia, a middle incision was performed, and bowel distention was initially noted due to a 7x6x6 cm obstructing mass. The tumor was arising from the small bowel 70 cm away from the ileocecal valve. A second mass, 3 cm in diameter, which protruded inside the small bowel’s lumen, was also identified as 4 cm caudally from the initial mass. The masses did not infiltrate any other organs. A resection of the involved segment of the small bowel (about 35 cm), along with its mesentery, was performed (Figure 3). The segment included both masses within oncological margins. A side-to-side, stapled, small bowel anastomosis was performed. The patient had an uncomplicated course and was discharged on postoperative day three.

**Figure 4:**
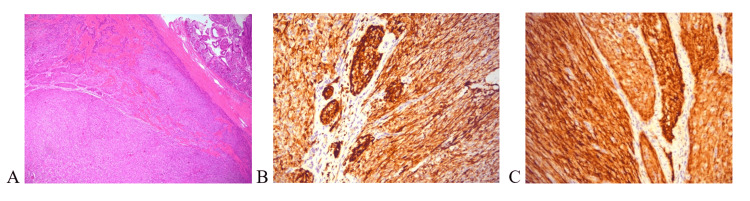
Immunohistochemical staining reports of the patient showing a malignant peripheral nerve sheath tumor (A), desmin (B), and CD56 (C).

Histopathological examination and extensive immunohistochemical staining confirmed the diagnosis. Both lesions had identical morphology. The histopathological report revealed mesenchymal tumors composed of spindle cells arranged partially in wavy hypercellular fascicles with regional necrosis and mitotic activity of about 10-14 mitoses/hpf, as well as hypocellular areas with myxoid stroma. Perineural encasement was evident. The neoplasm scored as intermediate-grade; immunohistochemistry expression for both lesions showed positivity for desmin, muscle actin, CD56, CD99, TLE-1, and Ki67 at 15%. The tumors were negative for AE1/AE3, CK19, p63, S-100, GFAP, D2-40, CD34, CD117, DOG-1, MDM-2, CDK4, h-Caldesmon, synaptophysin, chromogranin, HMB45, and MyoD1 (Figure 4). In conclusion, the diagnosis of MPNSTs was made.

Later, molecular testing of 86 different fusions was conducted for EWSR1, CIC, BCOR, SS18, SS18L1, PAX3, PAX7, TFE3, NTRK3, ETV6, HMAG2, OLAG1, JAZF1, MEAF6, HEY1, GLI1, TFG, ALK, FUS, CO1A1, and USP6 genes in our patient’s blood samples. However, the analysis using the sarcoma research assay panel was negative for mutations.

After a definite diagnosis, the multidisciplinary team's decision was that the patient should be treated as an intra-abdominal sarcoma patient, which included radiotherapy and close follow-up. Three months postoperatively, the patient underwent a CT scan without any indication of any recurrent lesions.

## Discussion

According to the literature, MPNSTs are extremely rare soft-tissue sarcomas, showing neuroectodermal differentiation with an aggressive nature that arises from either a peripheral nerve or any tissue that demonstrates peripheral nerve differentiation upon histopathological examination [2]. Interestingly, there is a high rate of local recurrence and hematogenous distant metastasis. It is commonly associated with NF1, as up to 50% of the patients with MPNSTs also have NF1. Usually, the medical history of these patients reveals radiation exposure. Patients’ age group varies between 20 and 50 years old, although patients with NF1 are diagnosed at a younger median age, without showing a significant difference in long-term survival [1, 2]. Malignant peripheral nerve sheath tumors usually arise from the trunk, the neck and head area, or the extremities. There are very few cases of MPNSTs in the gastrointestinal system, less than 20 worldwide, in which the tumor is considered to arise from the Aurbach plexus, growing either exophytically or intraluminally [6].

Malignant peripheral nerve sheath tumors can be elusive in their diagnosis due to their non-specific signs and symptoms. Patients may experience fatigue, abdominal pain, weight loss, vomiting, gastrointestinal bleeding, and rarely obstruction [3-8]. Due to this vague symptomatology, the preoperative diagnosis of MPNSTs can be quite difficult to establish [3, 5]. This was also the case with our patient, in which, due to the position of the mass, a preoperative biopsy was not feasible.

Early imaging should be considered in identifying a suspicious mass. Computed tomography scans and MRIs can be useful in the initial diagnosis of the tumor. Usually, MPNST presents as a large mass (over 5 cm in diameter), with irregular margins and intratumoral lobulation. On imaging, it is challenging to differentiate MPNST from other benign or malignant lesions. A malignant peripheral nerve sheath tumor has a heterogeneous T1 signal intensity on MRI due to the frequent regions of necrosis inside the tumor [1]. Should the patient present with intussusception, the “target sign” is visible in the U/S and the CT scan of the abdomen, but these cannot identify the nature of the tumor [6]. Lastly, a PET-CT scan may be used to detect any tumor metabolic activity [4].

Definite diagnosis of MPNST can be confirmed after histopathological examination based on morphological and immunochemical characteristics of the whole lesion. Malignant peripheral nerve sheath tumors are mostly spindle cell neoplasms with a fascicular growth pattern, proliferation of the subendothelial zone, and a great presence of neurofibromatosis components [6]. It is a heterogeneous neoplasm with multiple areas of necrosis, varying mitotic rates, and plural histological and differentiation patterns. Furthermore, high levels of p53 and Ki67 may be present [4]. There are no specific histological markers for MPNSTs; exclusion of leiomyosarcomas, gastrointestinal stromal tumors (GISTs), fibrosarcomas, or other similar pathologies is achieved by extensive immunohistochemical investigation [2].

If feasible, a preoperative biopsy and a histopathological report can be useful in identifying the nature of a mass preoperatively. However, due to the severe abdominal symptoms in our patient and the location of the tumor, an exploratory laparotomy was decided. This was also noted in other published cases that were diagnosed post-operatively, as most of them presented with intussusception or ileus [3-6, 8].

Due to the rarity of these cases, randomized trials cannot be easily designed. However, according to some meta-analyses of published cases, the most advocated action against MPNSTs is a wide surgical resection to remove the tumor en bloc with the surrounding tissue, ensuring clear surgical margins [7, 8]. In cases where surgery was not decided or clear margins were not achieved, adjuvant radiotherapy and chemotherapy were offered to the patients. However, the role of these treatments is controversial, as studies haven’t shown that there is an overall survival benefit through radiation and chemotherapy [3]. Additionally, radiotherapy might be difficult in cases of MPNSTs in the small bowel due to its location inside the abdomen. In extremely rare cases where the diagnosis of MPNST is made before surgery, neoadjuvant radiotherapy or chemotherapy should be considered prior to surgery. There are no trials or studies about unresectable or metastatic disease.

## Conclusions

To date, MPNSTs of the small bowel are a rare condition, with a difficult diagnosis workout and multiple treatment pathways. Resection and extensive immunohistochemical tests lead to a definite diagnosis. Due to the fact that there is no standardized management on the small bowel MPNSTs yet to be confirmed, a multidisciplinary approach should be initially considered. Complete resection of the tumor, radiotherapy or chemotherapy, and very close postoperative follow-up are currently the common practices for MPNST patients.
